# PIPINO: A Software Package to Facilitate the Identification of Protein-Protein Interactions from Affinity Purification Mass Spectrometry Data

**DOI:** 10.1155/2016/2891918

**Published:** 2016-02-07

**Authors:** Stefan Kalkhof, Stefan Schildbach, Conny Blumert, Friedemann Horn, Martin von Bergen, Dirk Labudde

**Affiliations:** ^1^Department of Proteomics, Helmholtz Centre for Environmental Research-UFZ, 04318 Leipzig, Germany; ^2^Department of Bioanalytics, University of Applied Sciences and Arts of Coburg, 96450 Coburg, Germany; ^3^Department of Applied Computer Sciences & Biosciences, University of Applied Sciences Mittweida, 09648 Mittweida, Germany; ^4^Institute of Clinical Immunology, Medical Faculty, University of Leipzig, 04103 Leipzig, Germany; ^5^Fraunhofer Institute for Cell Therapy and Immunology, 04103 Leipzig, Germany; ^6^Department of Metabolomics, Helmholtz Centre for Environmental Research-UFZ, 04318 Leipzig, Germany; ^7^Department of Chemistry and Bioscience, Aalborg University, 9220 Aalborg, Denmark

## Abstract

The functionality of most proteins is regulated by protein-protein interactions. Hence, the comprehensive characterization of the interactome is the next milestone on the path to understand the biochemistry of the cell. A powerful method to detect protein-protein interactions is a combination of coimmunoprecipitation or affinity purification with quantitative mass spectrometry. Nevertheless, both methods tend to precipitate a high number of background proteins due to nonspecific interactions. To address this challenge the software Protein-Protein-Interaction-Optimizer (PIPINO) was developed to perform an automated data analysis, to facilitate the selection of bona fide binding partners, and to compare the dynamic of interaction networks. In this study we investigated the STAT1 interaction network and its activation dependent dynamics. Stable isotope labeling by amino acids in cell culture (SILAC) was applied to analyze the STAT1 interactome after streptavidin pull-down of biotagged STAT1 from human embryonic kidney 293T cells with and without activation. Starting from more than 2,000 captured proteins 30 potential STAT1 interaction partners were extracted. Interestingly, more than 50% of these were already reported or predicted to bind STAT1. Furthermore, 16 proteins were found to affect the binding behavior depending on STAT1 phosphorylation such as STAT3 or the importin subunits alpha 1 and alpha 6.

## 1. Introduction

Proteins are team players. Virtually all protein functions are triggered, controlled, modulated, and conducted by protein complexes. Deregulation of protein complexes is the cause of many diseases as being documented, for example, cervical cancer, bacterial infection, leukemia, neurodegenerative diseases, and Huntington disease [1]. Consequently, the directed modulation of protein interactions is one of the upcoming fields in pharmacology and drug design [2, 3].

Unfortunately, the information which protein complexes are formed and are present at certain conditions can not directly be obtained from transcriptome or genome data. Thus, the comprehensive analysis of protein-protein interaction networks (interactome) and its quantification and dynamics are one of the most important issues in the postgenomic era.

In the last decades protein interactions were intensively investigated using small as well as large scale approaches. Much of the data is available and has been integrated in protein-protein interaction (PPI) databases such as Biological General Repository for Interaction Datasets (BioGRID) [4], the molecular interaction database (MINT) [5], the Biomolecular Interaction Network Database (BIND) [6], Mentha [7], the Database of Interacting Proteins (DIP) [8], the IntAct molecular interaction database (IntAct) [9], and the Human Protein Reference Database (HPRD) [10]. To prevent an enrichment of false positive interactions all database systems claim to use strict quality filters. At present, more than 50,000 nonredundant human PPIs are listed in at least one of the main public repositories. Interestingly, the overlap between the databases is still very small and many of the binary interactions are only listed in a single database. Furthermore, the recently updated commercial Prolexys Human Protein Interaction Dataset (Hynet) claimed to contain more than 300,000 experimentally determined human PPIs (unpublished data). Thus, one might conclude that either all public databases are still far from being comprehensive and/or that the databases still contain a huge amount of false positives.

One of the most powerful methods for small and large scale PPI studies is affinity purification or coimmunoprecipitation combined with mass spectrometry [11–13]. However, one has to be aware that there are several sources for the detection of false positive interaction partners. The curation of the obtained data is time consuming and thereby expensive. Therefore it is desirable to reduce the number of false positives to a minimum to spare resources and identify valuable interaction partners.

To experimentally identify true novel protein-protein interaction partners it is important to carefully conduct the PPI experiments including proper controls as well as to evaluate the experiments in respect to high quality data from previous studies. The data analysis should include statistical analysis of the raw data, data evaluation in respect of known contaminants such as beads binding proteins, and the recovery of already observed or predicted interaction partners. Additionally, putative PPI data can be filtered using functional information or correlation to large scale protein interaction networks. However, usually data analysis programs aim to filter the experimental data either using control experiments or by integration of functional information.

The software PIPINO (Protein-Protein Interaction Optimizer, http://www.bioforscher.de/pipino) is a novel attempt to integrate and combine the strengths of both approaches. PIPINO allows standardizing the data analysis process and offers a semiautomatic analysis pipeline. Beside various statistical methods for evaluating the data the software is capable of functionally annotating and enriching/filtering data entries with additional information. This refinement is accomplished by the use of curated interaction databases. Thus, a comprehensive interaction network can be created and used for the data analysis of a specific protein of interest. Database information and reliabilities in form of database scores for corresponding interactions can be used to assess the probability of the correctness of an interaction within the network through a normalized score as well as a visual representation. Additionally, it is possible to analyze and visualize perturbation of a network as it might be triggered by, for example, a bait phosphorylation. The performance of the software is demonstrated for the analysis of the interactome of the signal transducers and activators of transcription 1 (STAT1) with and without phosphorylation dependent activation. PIPINO is available at http://www.bioforscher.de/pipino.

## 2. Material and Methods

### 2.1. Generation of the Experimental PPI Dataset

#### 2.1.1. Plasmid Construction

An expression vector of biotinylated STAT1 was prepared as has been descripted recently for STAT3 [14]. Briefly, Rc/CMV-STAT1-Bio was constructed by cloning the human STAT1 cDNA into the EcoRI and SalI sites of pBluescript II KS (−). Afterwards the stop codon was replaced with a BamHI site by site-directed mutagenesis and a 23-amino acid carboxy-terminal biotinylation tag was added. Finally a Bsu36I-ApaI fragment of pB-STAT1-Bio was subcloned into the respective sites of Rc/CMV-STAT1. Thus, the expression vector Rc/CMV-STAT1-Bio was obtained. A BirA mammalian expression vector was constructed by subcloning the BirA cDNA into the EcoRI and Xho I sites of pBluescript II KS (−) (Stratagene, Heidelberg, Germany). Subsequently, the cDNA was inserted into the KpnI and XbaI sites of the expression vector pcDNA 3.1+ (Invitrogen, Karlsruhe, Germany). The expression vector for GFP-Bio was prepared as descripted [14].

#### 2.1.2. Pull-Down of Proteins with Streptavidin Beads

Ultralink streptavidin beads (from Thermo Fisher scientific, Waltham, USA) were washed with cell lysis buffer. After this equilibration step 50 *μ*L beads were incubated with cell lysates containing 3 mg of total protein to precipitate the biotinylated proteins including their interaction partners. After incubating the beads 1.5 hours at 4°C on a rotating platform the beads were washed 3 times with cell lysis buffer, and bound proteins were eluted by boiling 3 minutes with 50 *μ*L SDS sample buffer.

#### 2.1.3. SILAC Sample Preparation

For the investigation of activation dependent STAT1 interactions, three independent biological replicates were analyzed in two technical replicates. Therefore cell lysates of STAT1-Bio expressing cells, which were either treated with erythropoietin or left untreated, were compared. As a control, streptavidin pull-downs were performed from whole cell extracts of cells expressing either GFP-Bio and used as a third group.

In detail, cells were grown in SILAC Dulbecco's minimal essential medium (SILAC DMEM, (PAA, Pasching, Austria)) without lysine and arginine, supplemented with 10% dialyzed fetal calf serum (PAA, Pasching, Austria) and 1% penicillin/streptomycin (Invitrogen, Paisley, UK). 84 *μ*g/mL ^12^C-L-arginine and 146 *μ*g/mL ^12^C-L-lysine (both from Sigma-Aldrich, St. Louis, USA) were added to the “light” media while the same concentrations of  ^13^C_6_-L-arginine or ^13^C_6_^15^N_4_-L-arginine and ^2^H_4_-L-lysine or ^13^C_6_-^15^N_2_-L-lysine (Cambridge Isotope Laboratory and Invitrogen, resp.) were added to the “medium” or “heavy” media, respectively. After determining the time for full incorporation of the isotopic amino acids, HEK 293T cells were cultivated at least 6 days with SILAC medium before harvesting.

After washing the cells three times with PBS, whole cell extracts were prepared using modified RIPA buffer for affinity purifications (50 mM Tris-HCl, pH 7.8, 150 mM NaCl, 1% NP-40, 0.25% sodium deoxycholate 1 mM EDTA). Protease inhibitors 5 *μ*g/mL leupeptin, 5 *μ*g/mL aprotinin, and 1 *μ*g/mL pepstatin A were added freshly. Cells were incubated with RIPA buffer 10 min on ice, and extracts cleared by centrifuging for 10 min at 13,000 ×g and 4°C. Total protein concentrations were determined using a Bradford Assay.

For SILAC experiments, equivalent total protein amounts of light and heavy extracts were incubated separately with ultralink strep beads streptavidin beads for 1.5 hours. After 3 washing steps, bound proteins were eluted with SDS sample buffer, then combined, and subjected to SDS-PAGE.

#### 2.1.4. Protein Separation, Liquid Chromatography Tandem Mass Spectrometry, and Data Analysis

Samples from cells grown in heavy, medium, and light medium were mixed and 0.125 M Tris-HCl buffer containing 4% SDS, 20% (v/v) glycerol, 0.1% (m/v) bromophenol blue, and 10% (v/v) 2-mercaptoethanol was added. After heating the mixtures 5 min at 95°C the proteins were separated using 1D-SDS-PAGE (12%). After staining the proteins each lane was cut into 10 slices of approximately equal protein amounts. Proteins were destained and desalted within the gel slices and tryptic digestion was carried out using porcine trypsin.

All protein digestions were analyzed by nano-uHPLC/nano-ESI-MS/MS using a LTQ Orbitrap XL ETD (Thermo) online coupled via a chip-based nano-ESI source (Nanomate, Advion) to a nano-uHPLC (nanoAcquity UPLC, Waters Corporation, Milford, USA) as described before [15].

Mascot [16] (version 2.3.01, Matrix Science, London, UK) embedded in ProteomeDiscoverer (version 1.4, Thermo Sci.) was used to perform the identification and quantification of proteins. For the database search a concatenated target/decoy database which contains all correct as well as the reversed entries of the Swiss-Prot database species human [17] (http://www.expasy.org/, UniProt Consortium, 09-2010, 40924 forward and reverse sequences) was utilized. Thereby the protein and peptide false discovery rates were controlled to be below 0.05. For peptide identification up to a maximum of three isotope-labeled amino acids and maximum two tryptic missed cleavages were considered. A mass error of up to 0.5 Da for MS/MS product ions and up to 20 ppm for MS precursor ions was tolerated. Methionine oxidation, acetylation (protein N-terminus), asparagine and glutamine deamidation (all optional), and cysteine carbamidomethylation (complete) were considered as modification. On request the complete GeLC-MS raw data as well as details concerning identifications and quantifications will be provided. All raw quantitation ratios as well as the results of all filtering and statistical analysis steps of the three conditions are summarized in Supplementary Table 1 (see Supplementary Material available online at http://dx.doi.org/10.1155/2016/2891918).

### 2.2. Data Analysis Using PIPINO

#### 2.2.1. Overview

Data analysis was accomplished using the in-house software called “PIPINO” (Protein-Protein Interaction Optimizer). It is capable of visualizing and analyzing data and supports the selection of bona-fide interaction partners based on literature data such as protein interaction networks, frequency data, and bead proteome lists. The application is written in pure Java and is available as a standalone version including a detailed description at http://www.bioforscher.de/pipino.

#### 2.2.2. Data Upload

In this study MS raw data were processed and protein abundance ratios were calculated by ProteomeDiscoverer (Thermo Sci.). However, this is not a prerequisite for the application of PIPINO. In fact, initial quantitative proteomics data analysis by any other software or search engine can be used.

For a successful import of user data, the software requires a column separated data format containing experimental data (experiment descriptors, enrichment ratios) paired with general information regarding the experiment (protein of interest, UniProt accession numbers, gene names, and descriptors). The parser is flexible enough not to demand a special data format. The knowledge of the concrete data format and where needed information is located are sufficient to import data. This input system enables to use common shared data formats, for example, generated by ProteomeDiscoverer (Thermo Sci.), MaxQuant [18] (MPI Munich), Biotools (Bruker Daltonics), and user defined data formats.

#### 2.2.3. Parsing and Converting

User data is parsed and converted into a uniform intermediate data format understandable by the software. This can be achieved through a step-by-step transformation that utilizes regular expressions (RegEx). An example of a transformation can be found in the Supplementary Information.

First the document needs to be structured separating the header from the content and specifying field delimiters. The numbers of different experiments and samples per experiment (replicates) in the document are required in this step. The source columns of the user's document (characterized by the header fields) can be mapped to the target data model fields of the software. In this mapping either the whole data field or just a fragment specified through an extraction pattern in form of a regular expression can be used. A preview shows the outcome of the model conversion on a few data rows and indicates whether values have been calculated properly. If the transformation result is satisfying and valid the conversion parameters can be stored as a template for upcoming data imports of the same data source. Finally the complete experimental data will be converted to the intermediate data format and represented in a tabular form.

Insufficient entries (e.g., missing values, less frequent data) or known nonbinding partners can be removed via accession numbers or gene names in an optional filter process. Additionally, a predefined list of bead proteomes [12] and a list taken from the Protein Frequency Library (PFL) [19] are provided, which can be used to mark these entries in the current data. Further user defined lists can be applied as negative, positive, and general markers. These marker types can support the analysis process.

#### 2.2.4. Data Analysis

The enrichment ratio *r*_*i*,*j*_ and the probability value *p*_*i*,*j*_ are calculated for every protein *P*_*i*_ and experiment *E*_*j*_. These values are used as core features for data analysis purposes. A calculation starts with the value *V*_*i*,*j*,*k*_ of a corresponding experiment *E*_*j*_ and sample *S*_*j*,*k*_.

A precomputational step calculates the mean *S*_*j*,*k*_ (1) for each sample *S*_*j*,*k*_ over all proteins *P*_*i*_. In addition the median *V*_*i*,*j*_ (2) of all samples regarding a protein *P*_*i*_ and an experiment *E*_*j*_ is determined using the median operator M. Consider

(1) S−j,k=1m·∑i=1mVi,j,k,

(2) V~i,j=Mk=1nVi,j,k.

In the next step, the raw values *V*_*i*,*j*,*k*_ are log_2_-transformed and normalized with the help of the precomputed mean and median values (3). The normalization can be turned on and off by the user depending on the underlying data. Consider

(3) $$\begin{array}{c} V_{i,j,k}={log}_{2}\left(\;V_{i,j,k}\cdot {\tilde{V}}_{i,j}\cdot {{\overset{-}{S}}_{j,k}}^{-1}\;\right). \end{array}$$

Finally the ratio *r*_*i*,*j*_ (4) and the probability value *p*_*i*,*j*_ (5) can be calculated. For the probability value *p*_*i*,*j*_ a one-sided, paired *t*-test *T*(*x*, *y*) against a zero vector is utilized. Proteins with less than two samples for an experiment are considered as insufficient and therefore neither enrichment ratios nor probability values are calculated:

(4) ri,j=∑k=1nVi,j,kn,

(5) pi,j=TVi,j→,0→2with  Vi,j→=Vi,j,1⋮Vi,j,n,  0→=0⋮0.

#### 2.2.5. Data Visualization by Volcano Plot

As intermediate data is present, a visualization procedure can be initiated with the volcano plot to get an overview of the data distribution. The diagram applies the logarithmic enrichment ratio on the abscissa. The probability value (*p* value) can be interpreted as quality measure of the data and is applied on the ordinate. The logarithmic application of the enrichment ratio compensates the scattering of the data regarding the *p* values. Thus the plot is divided into a depleted part to the left of the ordinate and an enriched part to the right. As a result, all data points in the first quadrant of the diagram have been found enriched in the experiments. In general the more distant a data point can be found to the abscissa the more likely is its accuracy. As multiple experiments can be present in a single file, PIPINO is capable of switching between these experiments when displaying the volcano plot.

Furthermore, it is possible to highlight certain data points in the plot through their gene names or specifying thresholds on the data values for highlighting. In addition you can freely specify an area by adjusting thresholds for the ratio and the *p* value which results in a separation of the data points in the upper right area of the diagram. It is possible to select the separated data, as well as highlighted data or single data points. Selected entities are displayed in a detail table and may be exported to various data formats on demand for further investigation.

#### 2.2.6. Integration of Data from PPI Databases

PIPINO is capable of combining information from two different data sources, which can be categorized into the user defined input data of experiments and established interaction database inputs. The used interaction databases for information retrieval are listed in Table 1. IntAct, BioGRID, Mentha, and DIP encourage both the IMEx [20] standard and the PSI-MI standard [21] while covering a well curated interaction space. HPRD does not yet support the IMEx and PSI-MI standard but related to Mosca et al. [22] this database contains valuable and unique data regarding binary protein interactions. In addition the PIPs database is used to further enrich the interaction databases. The software is capable of including more interaction databases as required, respectively, to change the used databases according to specific needs.

A crucial factor for establishing a general network is the usage of mutual known identifiers. The UniProt accession number is chosen as a primary key for PPIs caused by its wide dissemination. Unfortunately there are still many proteins not natively assigned with a UniProt accession number. Therefore an ID mapping process was established to resolve as many interactions as possible, even from interactions that exhibit missing information with respect to the input format information. The dataset will be automatically completed as far as possible through a mapping of UniProt accession numbers and associated gene names as well as taxonomic information from the UniProtKB. If the refinement fails, the interaction will be discarded and is not integrated into the network.

Finally, all nonredundant interactions obtainable from these databases are merged together into an interaction network. This network is used as a basis for all upcoming considerations regarding validated protein-protein interactions and will be referred to as raw network. Due to the heavy resource load the network preparation step is currently not yet a functional module of PIPINO. Nevertheless, the latest prepared raw network is provided next to the standalone software until a suitable module can been offered.

#### 2.2.7. Data Visualization as PPI Network

Next to the visualization by volcano plot, there is another approach to visualize the intermediate data, which is based on a network structure, respectively, a tree structure. The perfuse visualization toolkit [23] (beta release 2007.10.21) is utilized for this visualization. As precondition a prepared network (raw network data) as well as prepared intermediate data needs to be specified. The network visualization takes the raw network data to span a network around a protein of interest with a user defined depth, the so-called focused network. Depending on the given depth more time for calculating the network is required and more resources are needed for displaying. This centered network does not yet contain any further information stated by the user. Certainly it can be enriched with the user data resulting in a specialized network containing relevant interactions taken from the experiment. To direct the focus closer to missing entities within the network a truncated network variant can be derived. The reduction of the network starts at the outer leaves and continues iteratively until the root (protein of interest) is reached. While truncating, confirmed interactions connecting a leave with a node (end point interactions) are removed. Therefore the truncated network only contains valuable information if unconfirmed proteins are present.

As soon as a network is ready to be displayed it is possible to choose between different network visualization methods to select the best matching focus for the desired intention. A dynamic network powered by a force field, for example, can identify interaction hubs while a static radial network provides a clear structured overview of the network depth. A hierarchic arranged network otherwise is more suitable to identify interaction pathways with the highest comfort. The edges of the network indicate the overall scoring from the interaction databases and therefore can be used as a measure of the interaction reliability. Alternatively a tabular view of the network can be requested showing all available information column-wise and sortable.

The network nodes and leaves are colored regarding the status of an entry. As a result, it is possible to distinguish between data entries occurring only in the network, only in the intermediate data, and in both datasets. Export functionality can be used to generate lists of filtered proteins for further examination, for example, highlighting these proteins within the volcano plot.

## 3. Results

### 3.1. Workflow

**Figure 1:**
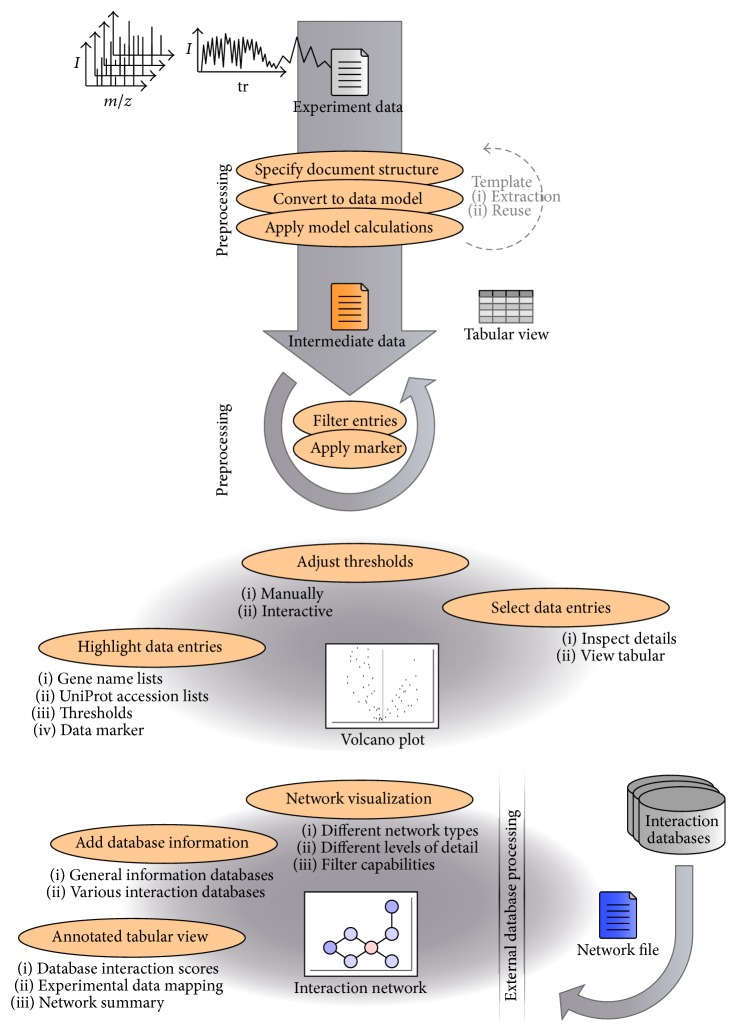
Utilization of PIPINO during preparation, data processing, and visualization of experimental AP-MS data.

The software PIPINO (Protein-Protein Interaction Optimizer) supports the analysis of AP-MS data by facilitating (i) raw data processing, (ii) interactive data visualization, (iii) comparison with data from PPI databases, and (iv) comparison with lists of proteins frequently observed in AP-MS experiments or known to bind nonspecifically and by providing additional network presentations (Figure 1). The current version of PIPINO is capable of dealing with a wide range of data from affinity purification mass spectrometry (AP-MS) experiments. In order to demonstrate the functionality and the handling of the software, this section outlines the application to the STAT1 interaction network.

### 3.2. Preexperiments for Analysis of the Interactome of STAT1 with and without Phosphorylation Dependent Activation

To map the STAT1 interactome with and without phosphorylation an AP-MS strategy was used as descripted recently in Blumert et al. [14]. It is based on in situ biotinylation of the bait protein to enable an efficient enrichment of bait/prey complexes and SILAC and allows the discrimination of potential false positives based on the relative protein quantities compared to a control AP-MS experiment.

The carboxy-terminus of STAT1 or GFP (for control) was fused to a 23-amino acid peptide tag carrying a target sequence for biotin protein ligases (biotag) and was coexpressed with the codon-optimized bacterial biotin protein ligase variant hBirA for in situ biotinylation in HEK 293T. Translation and biotinylation efficiency were monitored by Western blotting and visualized using streptavidin horseradish peroxidase conjugates. GFP-Bio and STAT1-Bio were found to be expressed in comparable amounts. For both proteins the biotinylation efficiency was not dependent on the amount of coexpressed hBirA or on the amount of biotin. Thus, it was considered to be stable and complete.

Cytokine-dependent tyrosine-phosphorylation and transactivation potential of either untagged STAT1 or STAT1-Bio were examined to verify that the STAT1 functionality was not affected by biotinylation. Because HEK 293T express only marginal levels of functional interleukin-6 receptor which is required for STAT1 activation, a chimeric receptor EG consisting of the extracellular domains of the erythropoietin receptor and the transmembrane and cytoplasmic parts of the interleukin-6 signal transducer gp130 was coexpressed, and cells were stimulated by adding erythropoietin (EPO) to the medium to activate STAT1. After EPO stimulation and overexpression of either STAT1 or STAT1-Bio the abundance of tyrosine-phosphorylation of STAT1 and STAT1-Bio was found to be equal.

### 3.3. AP-MS Analysis Resulted in 2221 Captured Proteins

A triple labelling SILAC strategy was applied to relatively compare the quantities of captured prey proteins between the control bait (GFP-Bio) and the target prey without activation (STAT1-Bio) and with EPO activation (pSTAT1-Bio). Therefore HEK 293T cells overexpressing GFP-Bio were labeled with “light” amino acids (Arg and Lys), cells expressing STAT1-Bio were labeled with “medium” amino acids (^13^C_6_-Arg and D_4_-Lys), and cells expressing STAT1-Bio and which were additionally stimulated with EPO were labeled with “heavy” amino acids (^13^C_6_^15^N_4_-Arg and ^13^C_6_^15^N_2_-Lys). An equal number of cells per condition were lyzed, bait/prey complexes were enriched by affinity chromatography, and the eluates were mixed. The combined eluates of the streptavidin pull-downs were separated by one-dimensional SDS-PAGE and the proteolytic peptides which were generated by tryptic in-gel digestion were analyzed using nano-HPLC/nano-ESI Orbitrap mass spectrometry. In total three biological replicates were measured in two technical replicates.

A data processing using the ProteomeDiscoverer revealed the identification and quantification of 2221 captured proteins (2 peptides, FDR < 0.05) in at least one of the six measurements. A file containing a list of all proteins including protein database identifiers and the relative quantification results between the three different channels (GFP-Bio, STAT-Bio, and pSTAT-Bio) was exported.

### 3.4. Parsing and Initial Processing with PIPINO

After parsing the data with an appropriate template (specified in the Supplementary Information) an initial data analysis can be conducted. In this step (i) technical replicates can be combined, (ii) optionally the biological replicates can be normalized and log_2_-transformed, and (iii) mean values and significance (*t*-test) are calculated. Furthermore, all proteins not quantified in at least three measurements (including technical replicates) were removed. Thus, in case of the STAT1 dataset 963 reproducibly quantified proteins (quantified in three replicates based on at least two peptides of which one was required to be unique) remained for further analysis once 1220 proteins have been sorted out.

### 3.5. Enrichment of Potential PPIs Based on Discarding of Typical False Positive Observed Proteins

Database information on proteins being frequently copurified such as endogenously biotinylated proteins (6 proteins) and typically bead binding proteins as well as proteins which are known to bind proteins involved in protein folding or degradation such as chaperons and proteases (19 proteins) was integrated and flagged to reduce the candidate list. The remaining 942 proteins were further investigated. Furthermore, a list of proteins which are frequently observed in numerous pull-down experiments as reported, for example, by Boulon et al. [19] and thereby assumingly represent unspecific binders was excluded as well.

### 3.6. Integration of PPI Database Information and Additional User Defined Data

Particularly for most of the human proteins but also for proteins of many other species there is information on known interaction partners listed in several databases. Automatic access to this information is highly valuable since this data can be used to judge the quality of the purification, to guide the following filtering steps, and to prevent redundant reporting of the so-called novel PPIs.

PIPINO allows the integration of all standard databases such as BioGRID [4], MINT [5], Mentha [7], DIP [8], IntAct [9], and HPRD [10] but also of costumer created databases. These databases were combined into a raw network comprising 1.451.141 valid but redundant interactions (Table 1). The network creation process merged redundant entries, removed interactions interacting with itself or with a protein with a different taxonomy identifier, and resulted in 498.345 nonredundant interactions between 79.369 different proteins. The PPI network of* Homo sapiens* comprises 201.110 interactions between 22.306 proteins.

This raw network was centered on STAT1 computed from the interaction databases MINT, IntAct, and Mentha entries for which interaction scores are available. The network was enriched by the altered experimental data to create a specialized network containing 17.568 proteins including 1240 proteins from the experiment, of which 1230 were part of the applied database. 10 proteins could not be found in the interaction databases. Further refinements of the network through truncation resulted in 1.575 remaining proteins. The possible interaction partners as well as the unconfirmed proteins in general can now be subjected for further analysis and studies.

For each of the filtered proteins (or if desired for all identified proteins) the information is provided whether and in which databases the proteins have been reported as bait binding partner.

### 3.7. Adjustment of Experimental Thresholds Using an Interactive Volcano Plot

**Figure 2:**
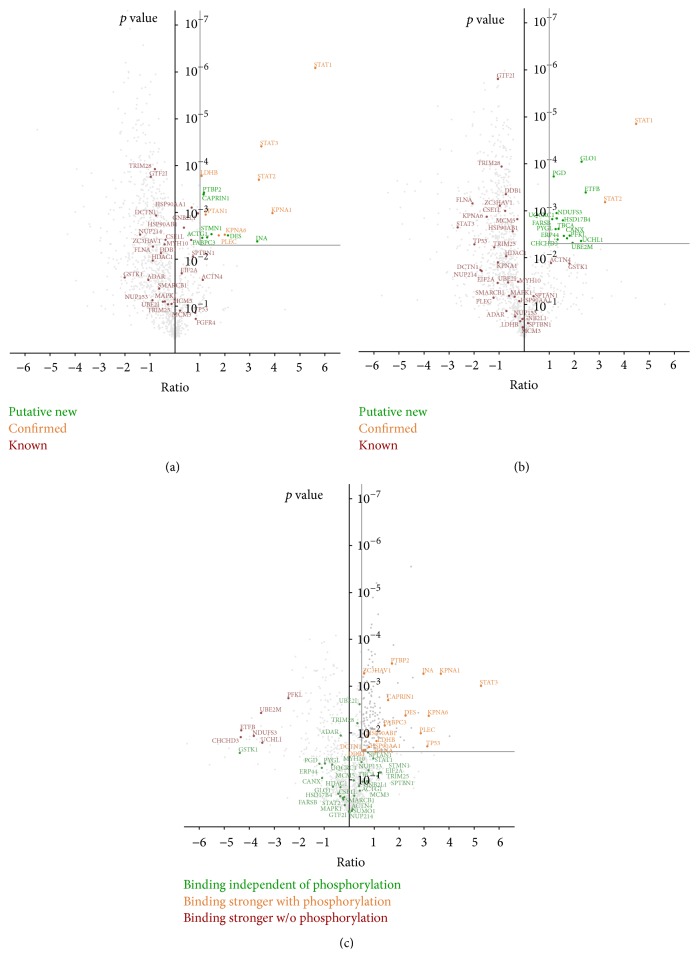
Volcano plots of results of the STAT1 AP-MS experiment. (a) The mean log_2_ ratios of STAT1-Bio with EPO treatment versus GFP-Bio are plotted versus the corresponding *p* values. X proteins are being depicted in red passing the thresholds of *p* value below 0.005 and log_2_-FC > 1 and were treated as putative STAT1 binding partners. (b) Analogously the volcano represents the AP-MS analysis of phosphorylated STAT1 versus GFP control. (c) Representation of the STAT1 activation experiments. Proteins which are identified as putative binding partners or which are derived as PPIs from literature are plotted depending on their binding properties to phosphorylated (enriched shown in blue) or nonphosphorylated (enriched shown in red) STAT1 proteins.

As an additional filtering step the data can be analyzed using the quantification information. In the STAT1 example the enrichment of all captured proteins is quantified compared to the pull-down of biotinylated GFP. The ratio of these quantities as well as *p* values can be utilized to further enrich potential protein interaction partners. The number of background proteins as well as the relative quantities of the captured binding partners can have high variations in AP-MS or Co-IP-MS experiments. Thus, to define and apply ratio or significance thresholds is not as straightforward as it is in standard proteomics applications. To support the definition of thresholds the processed data can be interactively visualized by the use of the integrated volcano plot as has been depicted in Figure 2. The volcano plot offers details on statistically significant entries corresponding to their median magnitude change throughout the experiment. The interactive handling of the plot enables to refine the thresholds and directly obtain information on the enrichment of known binding partners as well as on the depletion of background proteins (e.g., as listed in frequency libraries).

The volcano plots of either the STAT1-Bio versus GFP-Bio (Figure 2(a)) or the pSTAT1-Bio versus GFP-Bio (Figure 2(b)) reveal a clear separation of the data. Whereas the majority of the proteins are not significantly enriched, a minor fraction was observed with a log_2_ FC > 1 and a *p* value < 0.005. For the pSTAT1 dataset 15 proteins fulfill these additional criteria. With a proportion of > 50% well known STAT1 binding proteins are highly enriched. For the STAT1 dataset 17 proteins are part of the defined significance region of the volcano plot. Interestingly, only 2 of those have been already reported.

### 3.8. Network Analysis Reveals Information on Shared PPIs and Coverage of Known PPIs

**Figure 3:**
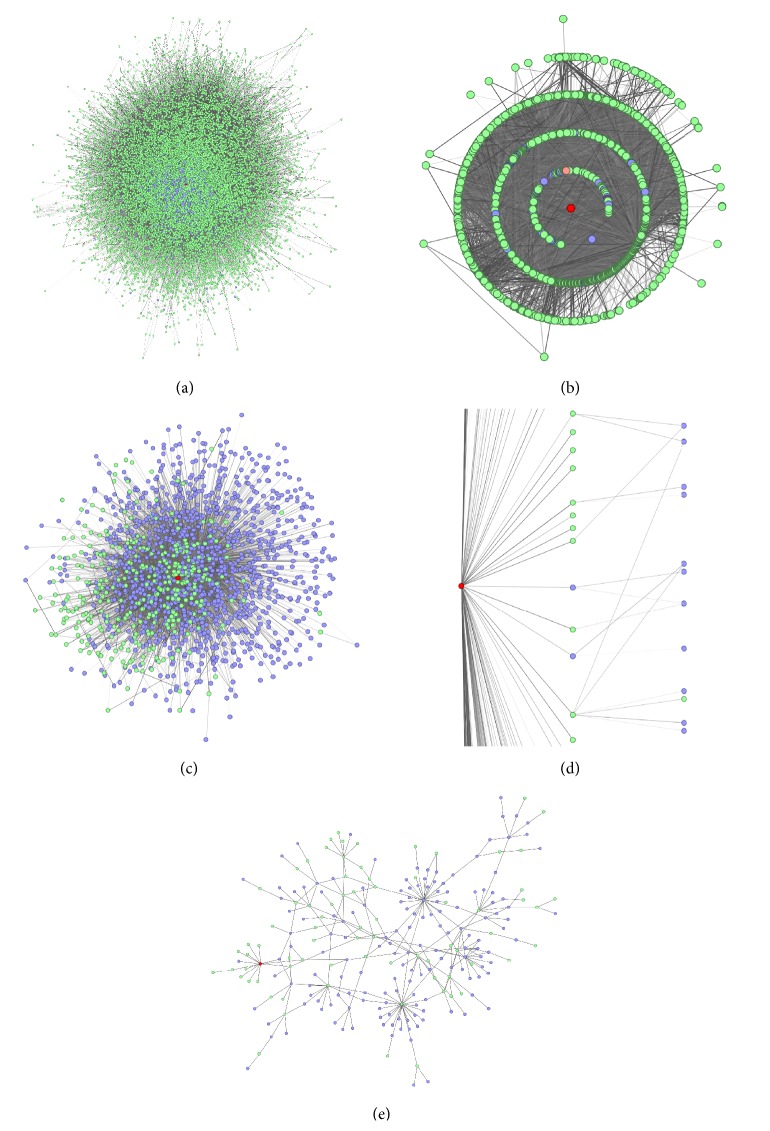
Different network layouts in different detail levels. (a) A specialized STAT1-centric network containing experimentally and literature derived PPIs arranged by a force field. (b) The same data depicted with a radial layout. (c) The corresponding truncated network arranged by a force field. (d) A detailed zoom-in of the hierarchical layout. (e) A truncated subnetwork with a scoring threshold of 0.85. Green nodes represent interaction partners not found in the experiment (only listed in database entries), the blue nodes are confirmed interaction partners (found in the databases and the experiment), and the red nodes represent the protein of interest.

At each time point and after every adjustment of a filtering criterion the result can be visualized using three different types of network presentations (Figure 3, cf.  Section 2.2.7). Furthermore, the amount of data which should be shown can be tailored. Whereas in the focused network only database information is shown, experimental and database information are combined in the specialized network (Figures 3(a) and 3(b)). Finally, the truncated network shows only the experimental proteins and their direct protein binding partners (Figure 3(e)).

The sophisticated overlay of interaction databases and experimental data helps to identify interaction pathways between proteins. It is possible to detect currently uninvestigated protein interactions as well as to identify possible protein hubs within the experiment. The information provided by the network analysis can be used to further encircle possible interaction partners while excluding irrelevant proteins.

In case of the pSTAT1 dataset using the truncated network proteins are visualized which either do directly interact with STAT1 or are only separated by one or two nodes (Figure 3(e)). These proteins are highly enriched after the filtering procedure, indicating that finally a highly purified bona-fide PPI list was obtained.

### 3.9. Phosphorylation Dependent Binding

Particularly the investigation of differences or dynamics in protein networks which are caused by drugs, toxic compounds, external stimuli, mutation, and so forth is an upcoming topic. In this study we investigated alterations caused by a cell treatment with EPO. EPO treatment caused a STAT1 phosphorylation, activation, and translocation to the nucleus. Thus one can expect a huge impact to the STAT1 interactome. Since all three conditions were processed and analyzed simultaneously it is possible to directly determine in a single analysis whether a protein binds STAT1 (enrichment in either the STAT1 or pSTAT1 pull-down compared to the GFP control pull-down) and if the same protein does bind differentially to STAT1. One protein which is well known to form heterodimers with STAT1 after phosphorylation but not without phosphorylation is STAT3 [24, 25]. As expected STAT3 was significantly identified as a pSTAT1 protein and was 5.3-fold enriched compared to the STAT1 pull-down.

A semiautomatic analysis revealed that in total 30 proteins were found to bind either STAT1 or pSTAT1 (Table 2). Of these proteins 16 were more than 1-fold enriched with a maximum value of 4.5. Of those 6 proteins were found to be much more enriched without activation whereas 10 were enriched after activation (Figure 2(c)). Other STAT1 interacting proteins which were found to be highly enriched are the Importin subunits alpha 1 and alpha 6, which are involved in the phosphorylation dependent nuclear import of STATs [26]. Furthermore, plectin and the lactate dehydrogenase B chain, two proteins which were predicted to bind to STATs, were found to be enriched. Other proteins which are enriched upon STAT1 phosphorylation are the filament proteins alpha-internexin and desmin, as well as the RNA binding proteins poly(A) binding protein, cell cycle associated protein 1, and the polypyrimidine tract binding protein 2.

Among the six proteins that showed stronger binding without activation are two proteins being involved in degradation (ubiquitin carboxyl-terminal esterase L1 and ubiquitin-conjugating enzyme E2M), two mitochondrial enzymes (electron-transfer-flavoprotein and NADH dehydrogenase (ubiquinone) Fe-S protein 3), the phosphofructokinase, and the coiled-coil-helix-coiled-coil-helix domain containing 3, which has been reported to be important for protein import in mitochondria but also to act as transcription factor and to regulate the BAG1 promotor. Interestingly, two known STAT1 binding partners the cytoskeletal protein spectrin [27] and especially STAT2, which is known to form STAT1/STAT2 heterodimers [28], showed no activation dependent binding.

## 4. Discussion

AP-MS is one of the most powerful approaches to identify protein interactions and can be applied for high-throughput studies. Several analysis tools have been designed to facilitate and automatize the identification of protein-protein interactions based on AP-MS data (recent review [29]) such as socioaffinity (SA) scoring [30], purification enrichment (PE) scoring [31], IDBOS [32], SAINT, CompPASS, or MiST. The scope of these approaches is to score and rank potential protein interaction partners based on data modelling. In case of SA, PE, and IDBOS, reliable modelling is based on large scale datasets, being created using the same AP-MS pipeline for dozens of baits. However, most of the experimental studies focus on the interactome of a low number or even single baits. For these low-throughput studies it is still a serious challenge to carefully control the false positive rate. The programs SAINT [33] and CompPASS [34] are applicable for small-scale sets. However, drawbacks of both programs are descripted by Teng et al. [29]. The CompPASS method was reported to perform well for large number of unrelated baits but seemed to filter out some true interactions with higher detection frequency when all baits belong to the same protein pathway. SAINT was observed to overpenalize true interactions, which were detected with high intensity but are not detected in all replicates.

Nevertheless, in addition to the computation of empirical or probabilistic scores (e.g., using CompPASS [34] or SAINT [33]) several bioinformatics tools can be applied and literature data can be included to successfully extract a reliable bona-fide interaction partner list from focused AP-MS analysis. As have been recently reviewed by Nesvizhskii [35] useful computational tools allow us to filter the lists of potential interacting partners based on fold changes and *p* values (e.g., using Perseus [36]) discarding likely false positive proteins (Decontaminator [37]), and interference analysis with predicted (e.g., by text mining or structural homology) or reported interaction data (e.g., using MINT, FunCoup [38], or STRING [39]). However, the different tools are independent and the results cannot be easily combined and visualized.

We concluded that especially in cases in which in-depth analysis of the protein interaction network of one or only a few selected baits is investigated it might be beneficial to automate the data processing and provide the researcher a maximum of additional data and visualization options. Based on these different types of information which are quantitative MS data, reproducibility of the AP-MS measurements, detection frequencies in previous experiments, biological functions, presence in PPI repositories, and so forth, the researcher has the possibility to define own thresholds and to report and prove the putative protein interactors.

Therefore PIPINO is designed for an initial processing of small datasets (few or even only one bait) by the use of interactive visualization and evaluation. The software allows a full processing starting with raw quantification data as has been exported by, for example, MaxQuant, ProteomeDiscoverer, PLGS, and Biotools, and is conducting all steps including data normalization, filtering based on fold changes, *p* values, and contaminant lists, an interference analysis with standard or manually curated PPI databases as well as pathway analysis within a bait centric network and the PPI data export. Particularly useful for a fast modification of data processing process is the fact that the results can be visualized as an iterative volcano plot or in three different network presentations.

Thus, the compact and user-friendly interface integrates all modules in a single application and is prepared for upcoming modules to be integrated as well. Therefore workflows and pipelines can be kept simple and straightforward. Due to the use of Java, the software can basically run on all operating systems and is not limited to Windows or Linux. Furthermore there is no complicated installation routine necessary, PIPINO can be started from within its folder, and therefore it is even possible to execute it as a portable application.

Using PIPINO the phosphorylation dependent STAT1 interactome studied by a single-step triple-SILAC based AP-MS approach was analyzed in detail. Starting from more than 2000 captured proteins it has been possible to finally extract a list of 30 potential interaction STAT1 partners of which more than 50% were already reported. Interestingly the semiautomatic analysis with PIPINO also revealed that 16 proteins were found to change the binding behavior depending on the STAT1 phosphorylation state such as STAT3 or Importin subunits alpha 1 and alpha 6.

## 5. Concluding Remarks

PIPINO can be used as an effective and supportive tool for analyzing protein-protein interactions obtained from experimental methods located in the field of affinity purification and mass spectrometry based quantitative proteomics. The software is capable of processing a large amount of data formats while enlarging the information space through curated interaction databases. Filtering, annotating, categorizing, and visualizing data entries, respectively, possible interaction partners for a protein of interest are available as a solid basis for interaction analysis.

Enhanced algorithms for network considerations, database handling, information extraction, and data refinement are currently under development and evaluation. These novel approaches will further increase the accuracy of current methods, enhancing the usability of the software and reducing needed efforts for the user in analyzing possible protein-protein interactions.

## Supplementary Material

I. Input parameter for the application to the STAT1 interaction network.
The header size of 1 and a single tabulator as field delimiter was chosen to structure the document. The amount of experiments was set to 3 with 6 samples per experiment. The accession number could be retrieved directly with the default extraction pattern “(.∗)”, while the gene name was extracted from the description field through the extraction pattern “GN=(.∗) P”. The taxonomy id was set manually to “9606” (Homo sapiens). The three experiments have been named manually and the associated sample values could be retrieved directly from appropriate fields. All adjusted parameters have been saved as a template for the ProteomeDiscoverer with three experiments and six samples and therefore can be loaded for upcoming parsing assignments.
Supplementary Table: AP-MS data of all proteins being quantified in this study. Additionally, for each protein the fallowing information are provided: (Quantification frequency, enrichment compared to control, prediction of STAT1 binding, function as chaperon or protease).

## Figures and Tables

**Table 1:** Interaction databases used to create a PPI netpwork. Databases marked with an asterix (*∗*) had been taken from the IMEx interface (http://www.ebi.ac.uk/intact/imex/main.xhtml) instead from direct database sources. An interaction was counted as invalid with one of the following reasons: it does not have a valid UniProt accession number or it could not be mapped to one, it has no valid gene name or it could not be mapped to one, or it has no valid taxonomy ID or it could not be mapped to one. Unfortunately, some mapping processes do not yet cover a large percentage of specific databases (cf. DIP^*∗*^ with 97% invalid interactions or InnateDB with 100% invalid interactions).

Name	Date	Interactions			IMEx
		Total	Invalid	Valid	
Mentha	17.08.14	461,408	47.236 (10%)	414.172 (90%)	*✗*
IntAct	18.07.14	424,706	128.680 (28%)	326.026 (72%)	✓
BioGRID	01.08.14	749,913	493.819 (66%)	256.094 (34%)	✓
MINT^*∗*^	26.03.13	122,356	17.968 (15%)	104.388 (85%)	✓
PIPs	12.09.08	34,216	9.466 (28%)	24.750 (72%)	*✗*
HPRD	13.04.10	39,240	29.288 (75%)	9.952 (25%)	*✗*
UniProt^*∗*^	18.08.14	11,919	2.272 (19%)	9647 (81%)	✓
DIP^*∗*^	07.01.14	107,619	104.050 (97%)	3.569 (03%)	✓
MPIDB^*∗*^	18.08.14	1,759	767 (44%)	992 (56%)	✓
I2D^*∗*^	18.08.14	1,117	263 (24%)	854 (76%)	✓
BHF-UCL^*∗*^	18.08.14	911	315 (35%)	596 (65%)	✓
InnateDB^*∗*^	17.08.14	680	93 (14%)	587 (86%)	✓
MatrixDB^*∗*^	18.08.14	1,244	864 (69%)	380 (31%)	✓
MolCon^*∗*^	18.08.14	495	129 (26%)	366 (74%)	✓
MBInfo^*∗*^	18.08.14	638	306 (48%)	332 (52%)	✓

**Table 2:** AP-MS data of all proteins being determined as putative interaction of phosphorylated and/or nonphosphorylated STAT1. Significant enrichments are highlighted in bold.

Gene name	Binding to phosphorylated STAT1			Binding to nonphosphorylated STAT1			Binding depending on phosphorylation			Known from literature
	STAT1 + EPO versus GFP			STAT1 versus GFP			STAT1 + EPO versus STAT1			
	log_2_ ratio	Standard deviation	*p* value (*t*-test)	log_2_ ratio	Standard deviation	*p* value (*t*-test)	log_2_ ratio	Standard deviation	*p* value (*t*-test)	
CHCHD3	−1.78	1.17	5.9*E* − 02	1.32	0.22	4.4**E** − 03	−4.35	1.20	2.4**E** − 02	No
ETFB	−1.48	0.58	2.3*E* − 02	2.45	0.12	4.1**E** − 04	−4.33	1.01	1.8**E** − 02	No
NDUFS3	−2.16	0.84	2.3*E* − 02	1.29	0.10	1.0**E** − 03	−3.82	1.02	2.3**E** − 02	No
UBE2M	−0.06	0.13	2.4*E* − 01	1.93	0.33	4.9**E** − 03	−3.53	0.54	7.7*E* − 03	No
UCHL1	−0.86	0.48	4.5*E* − 02	2.27	0.37	4.3**E** − 03	−3.48	1.10	3.2**E** − 02	No
PFKL	0.26	0.45	2.1*E* − 01	1.82	0.26	3.5**E** − 03	−2.44	0.26	3.7**E** − 03	No
PGD	−0.18	0.72	3.3*E* − 01	1.18	0.13	1.8**E** − 04	−1.20	0.97	9.1*E* − 02	No
ERP44	−0.49	0.43	5.3*E* − 02	1.58	0.47	3.4**E** − 03	−1.10	0.99	1.1*E* − 01	No
CANX	−0.40	0.45	8.6*E* − 02	1.70	0.53	3.9**E** − 03	−1.09	1.26	1.8*E* − 01	No
PYGL	−0.02	0.29	4.5*E* − 01	1.26	0.33	2.4**E** − 03	−0.97	0.79	9.0*E* − 02	No
UQCRC1	0.29	0.30	4.6*E* − 02	1.30	0.45	1.5**E** − 03	−0.68	0.70	9.4*E* − 02	No
GLO1	0.96	0.83	5.1*E* − 02	2.29	0.20	9.5**E** − 05	−0.66	1.00	2.8*E* − 01	No
HSD17B4	−0.06	0.39	4.1*E* − 01	1.54	0.36	1.7**E** − 03	−0.44	0.72	4.0*E* − 01	No
FARSB	−0.24	0.49	2.0*E* − 01	1.11	0.25	1.5**E** − 03	−0.37	0.83	4.4*E* − 01	No
STAT2	3.35	0.38	2.0**E** − 04	3.23	0.55	6.5**E** − 04	−0.24	0.65	5.2*E* − 01	Yes
ACTG1	1.08	0.61	3.6**E** − 03	0.34	0.24	1.6*E* − 02	0.42	0.86	3.4*E* − 01	No
TBCA	1.25	0.76	1.0*E* − 02	1.36	0.54	2.4**E** − 03	0.72	1.08	2.1*E* − 01	No
SPTAN1	1.22	0.25	1.1**E** − 03	0.37	0.36	6.6*E* − 02	0.75	0.48	5.1*E* − 02	Yes
STAT1	5.61	0.53	8.1**E** − 07	4.47	0.75	1.4**E** − 05	0.97	1.02	6.9*E* − 02	Yes
LDHB	1.06	0.30	1.7**E** − 04	−0.16	0.47	2.2*E* − 01	1.09	0.89	3.0**E** − 02	Yes
STMN1	1.45	0.19	3.0**E** − 03	−0.39	0.70	1.7*E* − 01	1.26	1.26	1.4*E* − 01	No
PABPC3	1.28	0.18	3.3**E** − 03	−0.59	0.43	7.2*E* − 02	1.43	0.29	1.3**E** − 02	No
CAPRIN1	1.13	0.28	4.1**E** − 04	−0.32	0.24	2.2*E* − 02	1.55	0.58	4.0**E** − 03	No
PTBP2	1.16	0.06	3.8**E** − 04	−0.63	0.02	1.3*E* − 04	1.71	0.07	6.1**E** − 04	No
DES	2.11	0.61	3.1**E** − 03	−0.30	0.48	1.5*E* − 01	2.25	0.73	8.6*E* − 03	No
PLEC	1.74	0.24	3.1**E** − 03	−1.23	0.89	7.0*E* − 02	2.86	0.71	2.0**E** − 02	Yes
INA	3.28	0.52	4.1**E** − 03	−0.32	0.49	1.9*E* − 01	2.97	0.17	1.1**E** − 03	No
KPNA6	1.99	0.26	2.9**E** − 03	−1.50	0.13	1.3*E* − 03	3.18	0.51	8.3*E* − 03	Yes
KPNA1	3.90	0.31	1.0**E** − 03	−1.06	0.29	1.2*E* − 02	3.67	0.58	1.1*E* − 03	Yes
STAT3	3.45	0.05	3.9**E** − 05	−2.67	0.31	2.2*E* − 03	5.28	0.40	1.9*E* − 03	Yes

## Abbreviations

PIPINO: Protein-Protein Interaction Optimizer
SILAC: Stable isotope labeling by amino acids in cell culture
PPI: Protein-protein interaction.
